# Supplementary figures and images for: Short-horizon neonatal seizure prediction using EEG-based deep learning
Source: PLOS Digit Health. 2025 Jul 11;4(7):e0000890. doi: 10.1371/journal.pdig.0000890 (PMC12250315; doi:10.1371/journal.pdig.0000890)

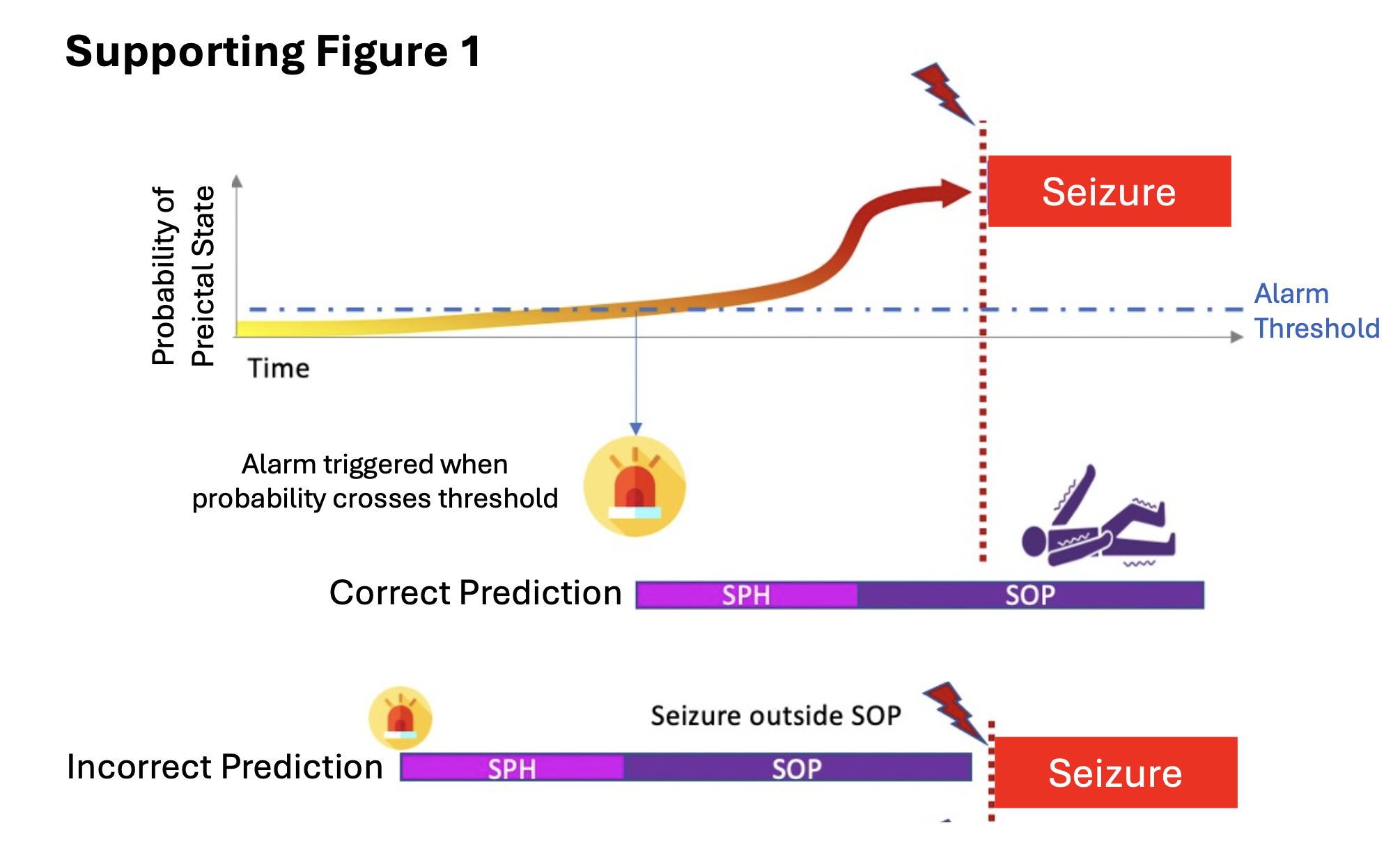

Supplement: S1 Fig — The seizure prediction horizon (SPH) and seizure occurrence period (SOP) evaluation framework as proposed by Maiwald et al. considers that there should be a minimum SPH to provide ample lead time before a seizure to allow for intervention and that the alarm should have SOP selected to align prediction duration with the specified clinical observation period. Following Maiwald et al., “the SOP is defined as a time period during which the seizure is to be expected” and the SPH “is a minimum window of time between the alarm raised by the prediction method and the beginning of SOP” (41). The system triggers an alarm, lasting the combined duration of SPH and SOP, if the designated seizure threshold is met. At time t, a true positive alarm occurs if a seizure initiates between t + SPH and t + SPH + SOP; otherwise, a false positive is marked. A false negative occurs if a seizure occurs at time ts and no alarm is activated. A true negative occurs when no alarm is triggered and no seizure occurs. (TIF) [file pdig.0000890.s001.tif]

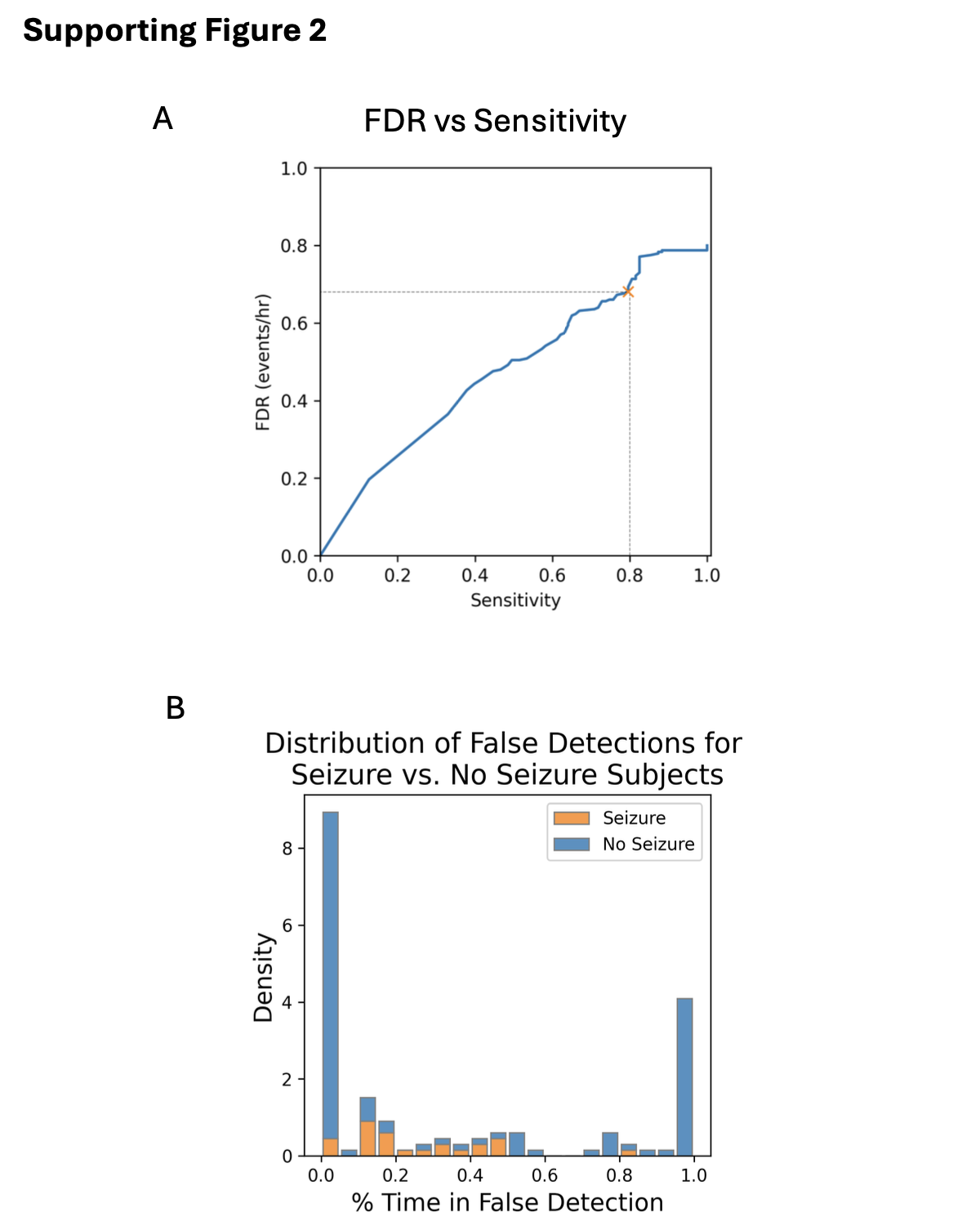

Supplement: S2 Fig — The relation between FDR and sensitivity is shown for ConvLSTM in the seizure prediction task with SPH of 3 minutes and SOP of 7 minutes. There is a resultant increase in FDR as sensitivity increases. The operating point corresponding with 80% sensitivity is denoted by the orange X, and this corresponds with an FDR of approximately 0.7. Details regarding sensitivity and FDR calculation are discussed in Methods, Seizure Prediction System Design and Evaluation Section, Paragraph 3. B. FDR was unevenly distributed across subjects, with certain subjects experiencing disproportionately higher FDR, particularly those without observed seizures. (TIFF) [file pdig.0000890.s002.tiff]

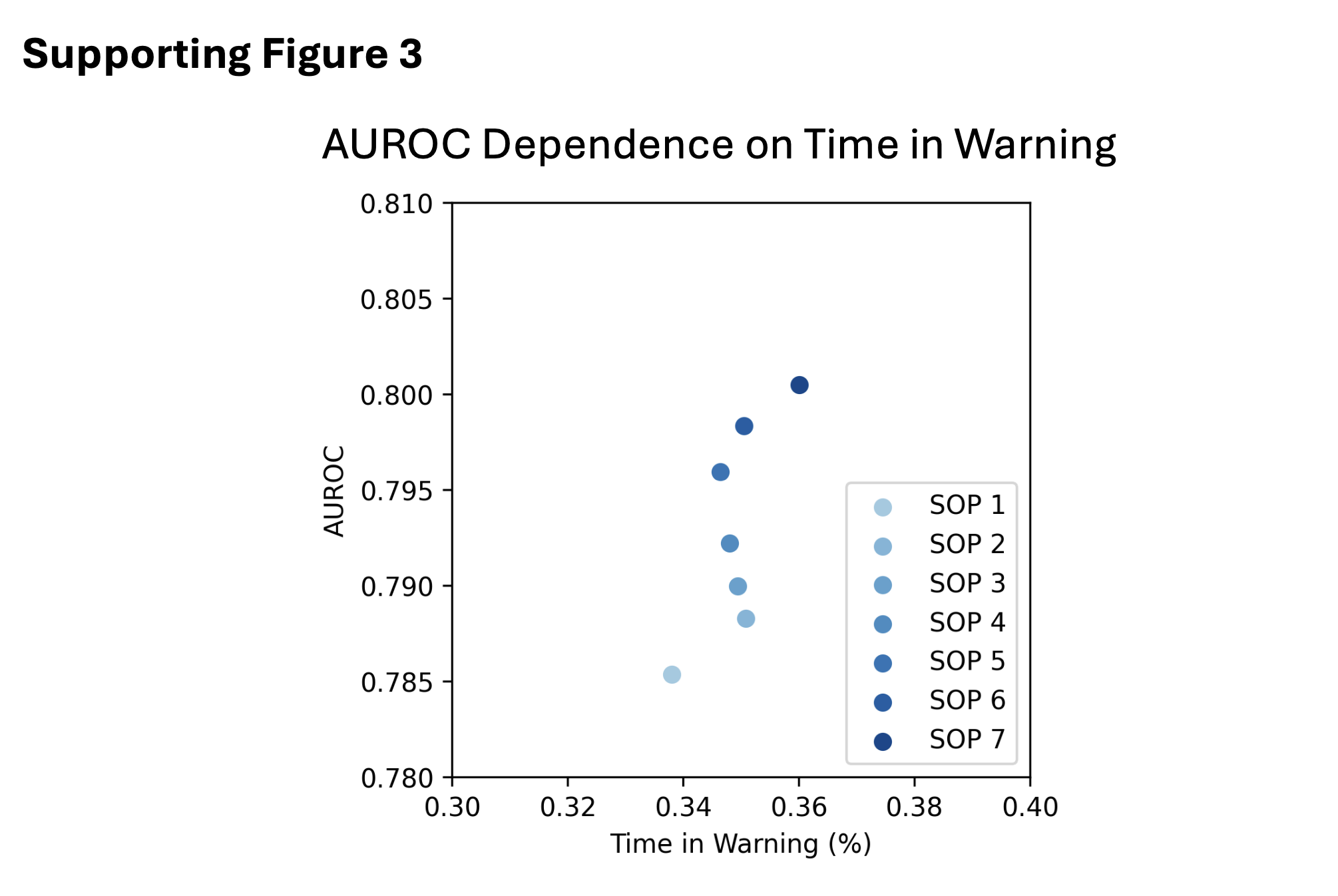

Supplement: S3 Fig — The relation between AUROC with Time in Warning (TIW) and Seizure Occurrence Period (SOP) is shown for ConvLSTM in the seizure prediction task with SPH of 3 minutes and varying SOP between 1–7 minutes. There is a resultant increase in AUROC and Time in Warning as SOP increases. (TIFF) [file pdig.0000890.s003.tiff]

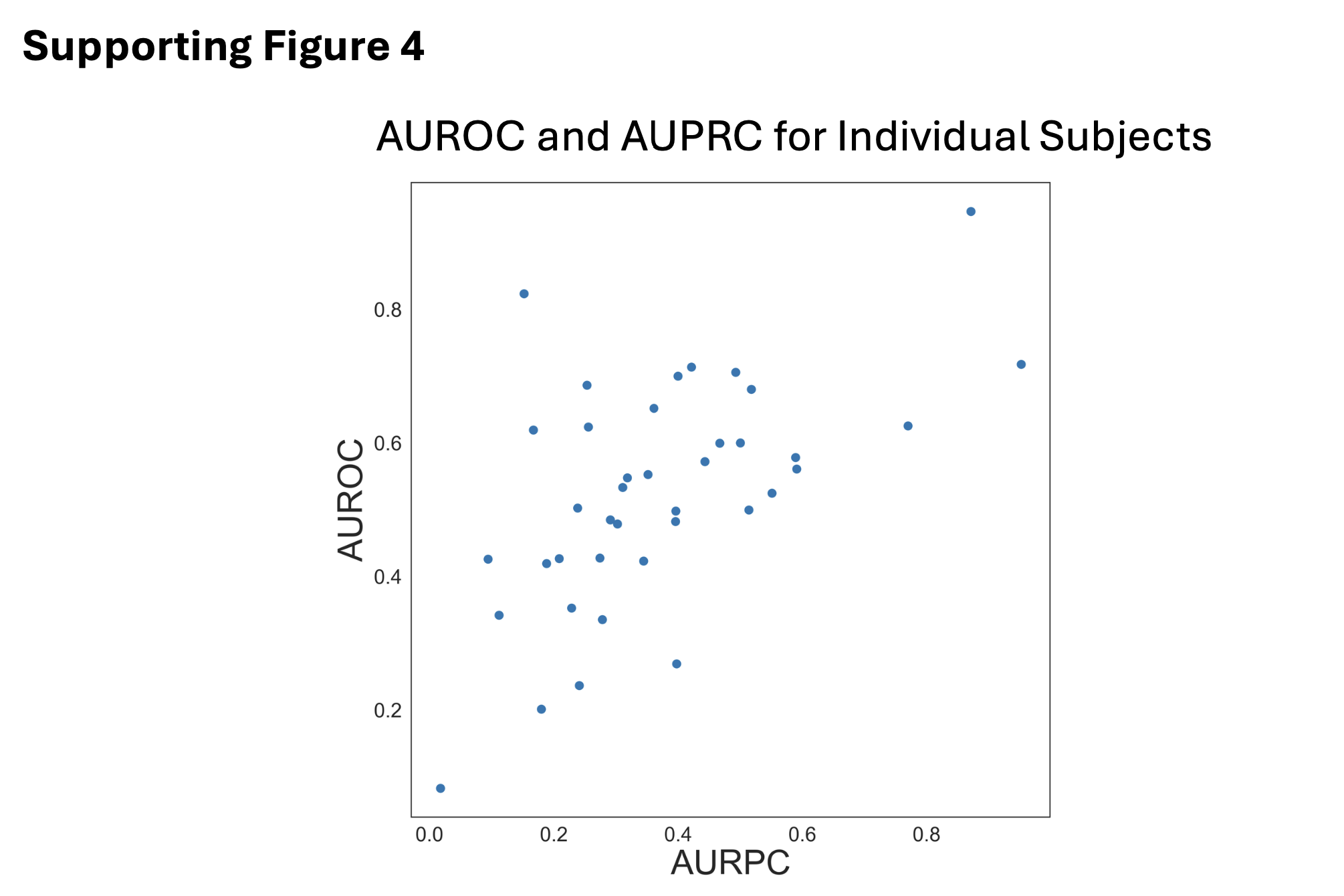

Supplement: S4 Fig — Individual AUROC and AUPRC scores are demonstrated on a per subject basis for all subjects who had seizures. There is no apparent clustering of subjects, and in general, performance AUROC and AUPRC appear correlated. Of note, only subjects who contained seizures were included in this analysis because AUROC calculation requires seizure occurrences for calculation of true and false positive rates. (TIFF) [file pdig.0000890.s004.tiff]

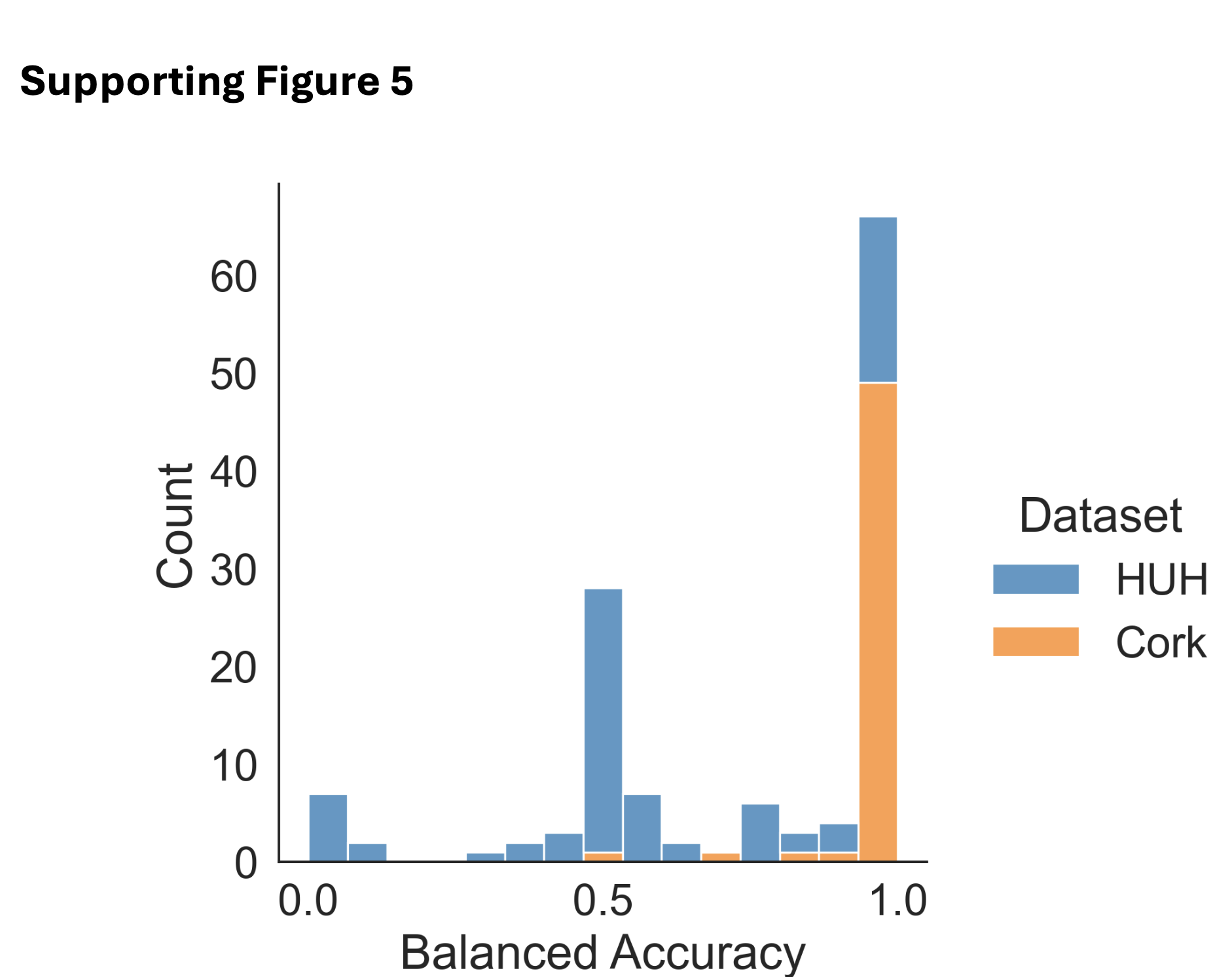

Supplement: S5 Fig — Preictal classification accuracy exceeded chance levels (50%) for most subjects, however, the performance was relatively higher in the Cork dataset compared to the HUH dataset. (TIFF) [file pdig.0000890.s005.tiff]
