# Supplementary material for: Short-horizon neonatal seizure prediction using EEG-based deep learning
Source: PLOS Digit Health. 2025 Jul 11;4(7):e0000890. doi: 10.1371/journal.pdig.0000890 (PMC12250315; doi:10.1371/journal.pdig.0000890)
Supplement: S1 Table — Asymmetry indices features were calculated across each montaged channel left-right pair, power spectral features calculated at each montage channel, and recurrence quantification analysis features calculated at each montage channel. (DOCX) [file pdig.0000890.s006.docx]

**S1 Table**

| Family | Abbreviation | Description |
| --- | --- | --- |
| Recurrence Quantification Analysis | RQA | We utilized features derived from Recurrence Quantification Analysis (RQA), a nonlinear data analysis technique based upon the recurrence plot, which is a plot of the recurrence of states or patterns in a time series (58). In each 20-second window, a recurrence plot was calculated for each montaged channel. The RQA features below (e.g. recurrence rate, determinism, laminarity, trapping time) were calculated on each per-channel recurrence plot. |
|  | RR | Recurrence rate |
|  | DET | Determinism |
|  | LAM | Laminarity |
|  | L_max | Longest diagonal line |
|  | L_entr | Entropy of diagonal lines |
|  | L_mean | Average diagonal line |
|  | TT | Trapping time |
| Asymmetry Indices |  | For each corresponding pair of channels, L and R, mirrored across the vertical axis, we calculate an asymmetry index utilizing the following formula: \|fn(L) - fn(R)\| / (fn(L) + fn(R)); 'fn' denotes a set of functions below (fn_mean, fn_std, fn_kurt, etc…) In this formula, 'fn' signifies a set of functions — (fn_mean) mean, standard deviation (fn_std), kurtosis (fn_kurt), skewness (fn_skew), the tenth percentile (fn_ten), and the ninetieth percentile (fn_ninety) — each of which is applied independently to the values of channels L and R. We then calculate the average of these function outcomes for each corresponding pair of channels. The goal is to quantify the differences between these mirrored (L/R) EEG channels. |
|  | Asymm_mean | Mean (fn_mean) |
|  | Asymm_std | Standard deviation (fn_std) |
|  | Asymm_kurt | Kurtosis (fn_kurt) |
|  | Asymm_skew | Skewness (fn_skew) |
|  | Asymm_p10 | 10th percentile (fn_ten) |
|  | Asymm_p90 | 90th percentile (fn_ninety) |
| Spectral power | Power_delta | Relative spectral power between 0.1 and 4 Hz |
|  | Power_theta | Relative spectral power between 4 and 8 Hz |
|  | Power_alpha | Relative spectral power between 8 and 12 Hz |
|  | Power_beta | Relative spectral power between 12 and 40 Hz |
