# Supplementary material for: Short-horizon neonatal seizure prediction using EEG-based deep learning
Source: PLOS Digit Health. 2025 Jul 11;4(7):e0000890. doi: 10.1371/journal.pdig.0000890 (PMC12250315; doi:10.1371/journal.pdig.0000890)
Supplement: S2 Table — (DOCX) [file pdig.0000890.s007.docx]

**S2 Table**

**EEG Dataset Characteristics^†^**

|  | **Cork**  **N=53** | **Helsinki**  **N=74** |
| --- | --- | --- |
| **Indication for Monitoring** |  |  |
| HIE | 53 | 35 |
| Other | 0 | 44 |
| **Clinical Characteristics** |  |  |
| Gestational Age (wks) | 40.0 (39.4 – 40.7) | 39.5 (37.75 – 40.5) |
| Birth weight (g) | 3,470 (3,190 – 3,800) | 3250 (2750 – 3750) |
| Hypothermia | 31 (58%) | - |
| **EEG Characteristics** |  |  |
| EEG Duration, total (hrs) | 169 | 111.9 |
| EEG Duration, per subject (hrs) | 3 (2 – 4) | 1.2 (1.1 – 1.6) |
| Subjects with seizures | 2 (4%) | 39 (52%) |
| Seizures per subject* | 2 (1 – 6) | 6 (2 – 12) |
| Median duration of seizures per subject* | 1.25 (0.63 – 1.8) | 1.2 (0.66 – 3.3) |

†Median values are reported under Clinical and EEG characteristics. The values in parenthesis indicate IQR or percentage. Abbreviations: hypoxic-ischemic encephalopathy (HIE), weeks (wks), grams (g), hours (hrs). Table adapted from clinical data from Stevenson et al. and O’toole et al (1, 2).

* Calculated across only subjects containing seizures

**References**

1. Stevenson NJ, Tapani K, Lauronen L, Vanhatalo S. A dataset of neonatal EEG recordings with seizure annotations. Sci Data. 2019;6:190039.
2. O’toole JM, Mathieson SR, Raurale SA, Magarelli F, Marnane WP, Lightbody G, et al. Neonatal EEG graded for severity of background abnormalities in hypoxic-ischaemic encephalopathy. Scientific Data. 2023;10(1):129.
