# Supplementary material for: Short-horizon neonatal seizure prediction using EEG-based deep learning
Source: PLOS Digit Health. 2025 Jul 11;4(7):e0000890. doi: 10.1371/journal.pdig.0000890 (PMC12250315; doi:10.1371/journal.pdig.0000890)
Supplement: S3 Table — (DOCX) [file pdig.0000890.s008.docx]

**S3 Table**

**K-fold Information Table**

| Fold Number | Epochs* per Fold | Hours per Fold |
| --- | --- | --- |
| 0 | 5806 | 32.3 |
| 1 | 5512 | 30.6 |
| 2 | 4814 | 26.7 |
| 3 | 5855 | 32.5 |
| 4 | 5066 | 28.1 |
| 5 | 6110 | 33.9 |
| 6 | 3275 | 18.2 |
| 7 | 4469 | 24.8 |
| 8 | 5090 | 28.3 |
| 9 | 3800 | 21.1 |
| Totals: | 49797 | 276.7 |

* Each epoch duration is 20 seconds and contains 14 EEG channels
