# Supplementary material for: Short-horizon neonatal seizure prediction using EEG-based deep learning
Source: PLOS Digit Health. 2025 Jul 11;4(7):e0000890. doi: 10.1371/journal.pdig.0000890 (PMC12250315; doi:10.1371/journal.pdig.0000890)
Supplement: S4 Table — The ConvLSTM-QEEG AUROC was compared to AUROC for other models using the Hanley McNeil test, and the differences in the AUROC were statistically significant between ConvLSTM and other architectures. In the task of preictal versus interictal state classification, ConvLSTM-QEEG (Table 1) generally outperformed DL methods utilizing automated feature extraction (AFE) directly on EEG, as presented here in Supplementary Table 4. Notably, certain DL models with AFE outperformed in metrics in isolation, with ConvLSTM-AFE obtaining higher AUROC (0.716), however, ConvLSTM-QEEG had the best performance across all metrics. It should be noted that some of the more recent time-series DL models (e.g., TSiT, OmniScaleCNN, and InceptionTime) were developed and validated on the UC Riverside Time-Series Classification dataset which, while expansive with some biological data including heartbeat and atrial fibrillation time-series, does not contain EEG data. Data reported as average performance across all cross-validation folds (10) with (standard error). Details regarding calculation of AUROC, AUPRC, and F1 are discussed in Supplementary Methods 3. (DOCX) [file pdig.0000890.s009.docx]

**S4 Table^†^**

**Comparison of DL methods using Automated Feature Extraction**

| Input size | Architecture | AUROC | AUPRC | F1 |
| --- | --- | --- | --- | --- |
| 10 sec | TSiT | 0.527 (0.018) | 0.089 (0.02) | 0.159 (0.032) |
|  | Transformer | 0.548 (0.044) | 0.101 (0.023) | 0.173 (0.032) |
|  | InceptionTime | 0.652 (0.034) | 0.131 (0.029) | 0.229 (0.038) |
|  | ResNet | 0.581 (0.043) | 0.108 (0.032) | 0.193 (0.041) |
|  | ConvLSTM-AFE | 0.580 (0.017) | 0.099 (0.023) | 0.19 (0.03) |
|  | OmniScaleCNN | 0.610 (0.030) | 0.111 (0.024) | 0.178 (0.039) |
| 20 sec | TSiT | 0.571 (0.041) | 0.111 (0.026) | 0.200 (0.043) |
|  | Transformer | 0.619 (0.044) | 0.139 (0.040) | 0.221 (0.047) |
|  | InceptionTime | 0.668 (0.022) | 0.128 (0.027) | 0.244 (0.037) |
|  | ResNet | 0.681 (0.035) | 0.148 (0.038) | 0.240 (0.044) |
|  | ConvLSTM-AFE | **0.716 (0.026)** | **0.153 (0.031)** | 0.240 (0.037) |
|  | OmniScaleCNN | 0.658 (0.050) | 0.143 (0.043) | 0.246 (0.050) |
| 40 sec | TSiT | 0.590 (0.043) | 0.112 (0.028) | 0.212 (0.050) |
|  | Transformer | 0.608 (0.045) | 0.129 (0.037) | 0.201 (0.043) |
|  | InceptionTime | 0.668 (0.035) | 0.129 (0.029) | 0.235 (0.040) |
|  | ResNet | 0.656 (0.033) | 0.142 (0.035) | **0.248 (0.052)** |
|  | ConvLSTM-AFE | 0.649 (0.031) | 0.137 (0.031) | 0.214 (0.039) |
|  | OmniScaleCNN | 0.674 (0.039) | 0.147 (0.042) | **0.248 (0.052)** |

**^†^**The ConvLSTM-QEEG AUROC was compared to AUROC for other models using the Hanley McNeil test, and the differences in the AUROC were statistically significant between ConvLSTM and other architectures. In the task of preictal versus interictal state classification, ConvLSTM-QEEG (Table 1) generally outperformed DL methods utilizing automated feature extraction (AFE) directly on EEG, as presented here in Supplementary Table 4. Notably, certain DL models with AFE outperformed in metrics in isolation, with ConvLSTM-AFE obtaining higher AUROC (0.716), however, ConvLSTM-QEEG had the best performance across all metrics. It should be noted that some of the more recent time-series DL models (e.g. TSiT, OmniScaleCNN, and InceptionTime) were developed and validated on the UC Riverside Time-Series Classification dataset which, while expansive with some biological data including heartbeat and atrial fibrillation time-series, does not contain EEG data. Data reported as average performance across all cross-validation folds (10) with (standard error). Details regarding calculation of AUROC, AUPRC, and F1 are discussed in Supplementary Methods 3. Abbreviations: Area Under the Receiver Operator Characteristic (AUROC), Area Under the Precision Recall Curve (AUPRC), Matthew Correlation Coefficient (MCC). F1 score (F1). Table adapted from Kim et al. (2025), Licensed under Creative Commons Attribution 4.0 International License (http://creativecommons.org/licenses/by/4.0/). Changes were made.
