# Supplementary material for: Short-horizon neonatal seizure prediction using EEG-based deep learning
Source: PLOS Digit Health. 2025 Jul 11;4(7):e0000890. doi: 10.1371/journal.pdig.0000890 (PMC12250315; doi:10.1371/journal.pdig.0000890)
Supplement: S5 Table — We benchmarked conventional ML methods which all utilized QEEG features and demonstrate that DL approaches generally demonstrated improved performance compared to ML methods. Data is reported as average performance across all cross-validation folds (10) with (standard error). Details regarding calculation of AUROC, AUPRC, MCC, and F1 are discussed in Supplementary Methods 3. (DOCX) [file pdig.0000890.s010.docx]

**S5 Table^†^**

**Comparison of conventional ML methods.**

|  | **AUROC*** | **AUPRC** | **F1** |
| --- | --- | --- | --- |
| Random Forest | 0.612 (0.021) | 0.147 (0.023) | 0.252 (0.030) |
| Support Vector Machine | 0.613 (0.037) | 0.163 (0.030) | 0.262 (0.031) |
| Logistic Regression | 0.598 (0.025) | 0.163 (0.027) | 0.243 (0.027) |
| K-Nearest Neighbors | 0.633 (0.022) | 0.112 (0.013) | 0.255 (0.029) |

**^†^**We benchmarked conventional ML methods which all utilized QEEG features and demonstrate that DL approaches generally demonstrated improved performance compared to ML methods. Data is reported as average performance across all cross-validation folds (10) with (standard error). Details regarding calculation of AUROC, AUPRC, MCC, and F1 are discussed in Supplementary Methods 3. Abbreviations: Area Under the Receiver Operator Characteristic (AUROC), Area Under the Precision Recall Curve (AUPRC), Matthew Correlation Coefficient (MCC). F1 score (F1). Table adapted from Kim et al. (2025), Licensed under Creative Commons Attribution 4.0 International License (http://creativecommons.org/licenses/by/4.0/). Changes were made.
