# Supplementary material for: Short-horizon neonatal seizure prediction using EEG-based deep learning
Source: PLOS Digit Health. 2025 Jul 11;4(7):e0000890. doi: 10.1371/journal.pdig.0000890 (PMC12250315; doi:10.1371/journal.pdig.0000890)
Supplement: S6 Table — (DOCX) [file pdig.0000890.s011.docx]

**S6 Table**

| **Year** | **Authors** | **Dataset** | **Feats** | **CLF** | **Same Cal** | **# Sz** | **Sens (%)** | **FPR (/h)** | **SOP (min)** | **SPH (min)** |
| --- | --- | --- | --- | --- | --- | --- | --- | --- | --- | --- |
| 2016 | Zhang & Parhi | MIT, 17 patients | power spectral density ratio | SVM | no | 80 | 98.68 | 0.05 | 50 | 0** |
| 2017 | Alotaiby et al | MIT, 23 patients | CSP | LDA | yes | 170 | 81 | 0.47 | 60 | 0 |
| 2018 | Khan et al | MIT, 15 patients | wavelet transform | CNN | yes | 18 | 83.33 | 0.15 | 10 | 0** |
| 2018 | Truong et al | MIT, 13 patients | short-time Fourier transform | CNN | yes | 64 | 81.2 | 0.16 | 30 | 5 |
| 2018 | Tsiouras et al | MIT, 23 patients | time domain, frequency domain, graph theory features, correlation features | LSTM | no | 185 | 100 | 0.06 | 30 | 0** |
| 2019 | Daoud & Bayoumi | MIT, 8 patients | DCAE + Bi-LSTM |  | yes | 43 | 99.6 | 0.004 | 60 | 0** |

**SPH implicitly set to 0 in these works

Abbreviations: Features (Feats), Classifier (CLF), Seizures (Sz), Sensitivity (Sens), Same Feature engineering (FE), Same Calibration (Cal) denotes studies which were calibrated on the same dataset used for training.
