# Supplementary material for: Short-horizon neonatal seizure prediction using EEG-based deep learning
Source: PLOS Digit Health. 2025 Jul 11;4(7):e0000890. doi: 10.1371/journal.pdig.0000890 (PMC12250315; doi:10.1371/journal.pdig.0000890)
Supplement: S1 Methods — (DOCX) [file pdig.0000890.s012.docx]

**S1 Methods**

**Machine Learning Comparison Models Methodology**

We utilized the python scikit-learn package to evaluate conventional machine learning (ML) methods including Support Vector Machine, K-Nearest Neighbors, Logistic Regression, and Random Forest classifiers. Training and evaluation was performed utilizing the same training, valid, and test datasets used to evaluate ConvLSTM. Hyperparameter optimization was performed using Optuna (1). The specific parameters considered were defined within the following ranges per model, with selected Optuna-suggested optimal parameters:

**ML Model Parameterization**

| **Classifier** | **Parameter** | **Options/Range** | **Selected** |
| --- | --- | --- | --- |
| Support Vector | C | 10^-5^ – 10^5^ | 1.2 |
|  | Kernel | linear, poly, rbf | rbf |
|  | Degree (for rbf) | 1 – 3 | 2 |
|  | Gamma (for rbf) | scale, auto | scale |
| Random Forest | Number of Estimators | 100 – 500 | 500 |
|  | Maximum Depth | 1 – 32 | 12 |
|  | Maximum Features | auto, sqrt | auto |
|  | Minimum Samples Split | 2 – 10 | 10 |
|  | Minimum Samples Leaf | 1 – 4 | 4 |
| K-Nearest Neighbors | Number of Neighbors | 1 – 30 | 3 |
|  | Weights | Uniform,  Distance | Uniform |
|  | Metric | Euclidean, Manhattan, Minkowski | Euclidean |
| Logistic Regression | Penalty | None, L1, L2, Elasticnet | L1 |
|  | C | 10^-4^ – 10^4^ | 0.25 |

**REFERENCES**

1. Akiba T, Sano S, Yanase T, Ohta T, Koyama M. Optuna: A next-generation hyperparameter optimization framework. InProceedings of the 25th ACM SIGKDD international conference on knowledge discovery & data mining 2019 Jul 25 (pp. 2623-2631).
