# Supplementary material for: Short-horizon neonatal seizure prediction using EEG-based deep learning
Source: PLOS Digit Health. 2025 Jul 11;4(7):e0000890. doi: 10.1371/journal.pdig.0000890 (PMC12250315; doi:10.1371/journal.pdig.0000890)
Supplement: S2 Methods — (DOCX) [file pdig.0000890.s013.docx]

**S2 Methods**

**Deep Learning Comparison Models Methodology**

We utilize the default time-series-ai (tsai) PyTorch framework to evaluate ConvLSTM, ResNet, Transformer, Time-series Transformer, InceptionTime, and OmniscaleCNN time-series deep learning models. Models were trained using one-cycle training (1) with initial learning rate of 0.001. 220 features per 20 second epoch were calculated, and the time duration of input was 5 minutes (15 total time steps or 5 * 3 twenty-second epochs), resulting in input tensor of dimensions 220 by 15 per sample. Regularization with drop-out was used, with the specific dropout rate used specified below. Normalization was performed where specified below. To address potential bias arising from class imbalance, loss weights were computed separately for each k-fold based on the relative proportions of pre-ictal and interictal segments. Following iterative hyperparameter optimization, we utilized the parameterizations below, following Kim et al. (2), Licensed under Creative Commons Attribution 4.0 International License (http://creativecommons.org/licenses/by/4.0/):

ResNet: We incorporate ResNet used as introduced by Wang et al. for time series classification (3), which is modified from the original version of ResNet by incorporation of 1-dimensional convolutional and pooling layers. We utilized 3 residual blocks, each with 3 convolutional blocks. We utilized convolution kernel sizes of 100, 20, and 3 samples.

ConvLSTM: The ConvLSTM architecture consisted of a convolutional layer with 256 output filters, followed by a batch normalization layer, then followed by an LSTM module with 256 cells and one hidden layer with size of 32, followed by a rectified linear unit (ReLU), followed by a 50% dropout layer, a fully connected linear layer (FCN), and then a final ReLU layer as final output. A softmax function was applied to the final ReLU layer to yield probabilistic predictions for each output class. For visualization purposes only 64 filters/cells are shown as opposed to 256.

Transformer (basic): Multiple variations of the original seminal Transformer (4) have recently emerged for time-series classification and prediction (5). We evaluate a basic, shallow encoder-decoder architecture to establish a baseline Transformer model. Model depth: 256, number of multiattention heads 8, number of sub-encoder-layers in the encoder: 3, number of sub-encoder-layers in the decoder: 3, dimension of the feedforward network: 256, activation: ReLU, epsilon value in normalization layers: 1e-5, dropout: 0.2.

Time Series Transformer (TSiT): TSiT is based on the Vision Transformer (6) and a similar approach has recently been utilized for eye movement classification from EEG (7). We utilized: Model depth: 12, multiattention heads 16, feedforward network dimension: 256, activation with GeLU, dropout: 0.1. To accommodate the long input sequences used (400, 800, 1600), we utilize a 1-dimensional convolutional layer with kernel size of 100 and stride of 50.

OmniscaleCNN: OmniscaleCNN utilizes multiscale 1d convolutional layers with a set of kernel sizes consisting of multiple prime numbers that varies in accordance with the length of the input time series (8). The resulting diverse set of receptive field configurations improves feature extraction by facilitating recognition of scale-invariant patterns. Subsequently, the architecture varies per input window length (e.g. 400, 800, 1600 for AFE). For example, the 800-input window length has in its first layer, 26 distinct OS-blocks, each characterized by differing kernel sizes, ranging from 1 to 97, and with batch normalization and ReLU activation. This is followed by a Global Average Pooling layer and concludes with a fully-connected linear layer.

InceptionTime: InceptionTime is based on the Inception architecture for image recognition and is comprised of ensembled CNNs (Inception modules) which extract both local and global time-series patterns (9). We utilized 5 Inception modules, with parameters of 32 filters and kernel size of 100.

**REFERENCES**

1. Smith, Leslie N. "A disciplined approach to neural network hyper-parameters: Part 1--learning rate, batch size, momentum, and weight decay." *arXiv preprint arXiv:1803.09820* (2018).
2. Kim J, Glass HC, Amorim E, Rao VR, Bernardo D. Comparison of Feature Engineering and End-to-End Machine Learning for Neonatal Preictal State Classification. InInternational Pediatric and Lifespan Data Science Conference 2025 (pp. 17-30). Springer, Cham.
3. Wang Z, Yan W, Oates T. Time series classification from scratch with deep neural networks: A strong baseline. In2017 International joint conference on neural networks (IJCNN) 2017 May 14 (pp. 1578-1585). IEEE.
4. Vaswani A, Shazeer N, Parmar N, Uszkoreit J, Jones L, Gomez AN, Kaiser Ł, Polosukhin I. Attention is all you need. Advances in neural information processing systems. 2017;30.
5. Zeng, Ailing, et al. "Are transformers effective for time series forecasting?." *Proceedings of the AAAI conference on artificial intelligence*. Vol. 37. No. 9. 2023.
6. Dosovitskiy A, Beyer L, Kolesnikov A, Weissenborn D, Zhai X, Unterthiner T, Dehghani M, Minderer M, Heigold G, Gelly S, Uszkoreit J. An image is worth 16x16 words: Transformers for image recognition at scale. arXiv preprint arXiv:2010.11929. 2020 Oct 22.
7. Yang R, Modesitt E. ViT2EEG: Leveraging Hybrid Pretrained Vision Transformers for EEG Data. arXiv preprint arXiv:2308.00454. 2023 Aug 1.
8. Tang W, Long G, Liu L, Zhou T, Blumenstein M, Jiang J. Omni-Scale CNNs: a simple and effective kernel size configuration for time series classification. arXiv preprint arXiv:2002.10061. 2020 Feb 24.
9. Fawaz, H. I., Lucas, B., Forestier, G., Pelletier, C., Schmidt, D. F., Weber, J. & Petitjean, F. (2019). InceptionTime: Finding AlexNet for Time Series Classification. arXiv preprint arXiv:1909.04939.
