# Supplementary material for: Short-horizon neonatal seizure prediction using EEG-based deep learning
Source: PLOS Digit Health. 2025 Jul 11;4(7):e0000890. doi: 10.1371/journal.pdig.0000890 (PMC12250315; doi:10.1371/journal.pdig.0000890)
Supplement: S3 Methods — (DOCX) [file pdig.0000890.s014.docx]

**S3 Methods**

Classification Model Performance Evaluation: We evaluated the Area Under the Receiver Operating Characteristic (AUROC), Area under the Precision-Recall Curve (AUPRC), F1 score and Matthew Correlation Coefficient (MCC) using the python sklearn package (1). For ConvLSTM probabilistic classification output was obtained, then under systematically varying decision thresholds, the AUROC was subsequently calculated using the true positive rate (TPR) and false positive rate (FPR) across these varying thresholds utilizing the sklearn Area Under the Curve (AUC) function which utilizes the trapezoidal rule. Additionally, we calculated AUPRC which is particularly informative in imbalanced datasets such as in this case, where interictal periods (negative class) significantly exceeds preictal periods (positive class) because it focuses on the model’s performance concerning the positive class. F1 score is also suited for model performance in the context of class imbalance and is the harmonic mean between precision and recall:

$$F1=2\times\frac{\text{Precision}\times\text{Recall}}{\text{Precision}+\text{Recall}}$$

1. Seizure Prediction Alarm System Performance Metrics

We calculated expected calibration error (ECE) utilizing provided python package following Gupta et al. (2). The formula for ECE is as follows:

$$\text{ECE}=\sum_{m=1}^{M} \frac{\left| B_{m} \right|}{N}\left| \text{acc}\left( B_{m} \right)-\text{conf}\left( B_{m} \right) \right|$$

where:

- *M* is the number of bins,
- *B_m_*​ is the set of indices of samples whose predicted probabilities fall into the *m*-th bin,
- *N* is the total number of samples,
- acc(*B_m_*​) is the accuracy of predictions in bin *m*,
- conf(*B_m_*​) is the average predicted probability (confidence) of the samples in bin *m*.

We calculated Brier Skill Score (BSS) under varying prediction horizons using the python sklearn package (1). The formula for BSS follows:

$$BSS=1 -\frac{BS}{BS_{\text{ref}}}$$

where:

- ​BS_ref_ is the reference climatological forecast.
- BS is the Brier Score for the predictions being evaluated, as follows:

$$\text{BS}=\frac{1}{N}\sum_{t=1}^{N} \left( p_{t}-o_{t} \right)^{2}$$

where:

- N is the number of predictions
- *p_t_* is the predicted probability
- *o_t_* is the actual outcome of the event at instance *t*

1. Pedregosa F, Varoquaux G, Gramfort A, Michel V, Thirion B, Grisel O, Blondel M, Prettenhofer P, Weiss R, Dubourg V, Vanderplas J. Scikit-learn: Machine learning in Python. the Journal of machine Learning research. 2011 Nov 1;12:2825-30.
2. Gupta C, Podkopaev A, Ramdas A. Distribution-free binary classification: prediction sets, confidence intervals and calibration. Advances in Neural Information Processing Systems. 2020;33:3711-23.
