# Supplementary material for: Short-horizon neonatal seizure prediction using EEG-based deep learning
Source: PLOS Digit Health. 2025 Jul 11;4(7):e0000890. doi: 10.1371/journal.pdig.0000890 (PMC12250315; doi:10.1371/journal.pdig.0000890)
Supplement: Attachment — Submitted filename: reviewer_comments.docx [file pdig.0000890.s016.docx]

**We deeply appreciate the insightful comments and feedback from the Reviewers. We address their feedback below, point by point. Major changes include:**

1. **Providing model training code on Github**
2. **Figure demonstrating subgroup analysis as requested by Reviewer 1**
3. **Figure demonstrating false detection distribution as requested by Reviewer 2**
4. **We have improved the clarity of Figures 3 and 4.**

**Reviewers' Comments:**

Reviewer #1:

Significant Comments

1. Part of the background discusses how a shorter interval EEG would be more feasible for resource poor countries. These deep learning technologies and the EEG data used for the study don’t seem feasible in these settings. How do you expect that this can translate to these populations.

We agree with the Reviewer that technology alone does not translate into clinical advances. We discuss this important point in the Limitations paragraph of the Discussion (line 282-285) as follows:

*“Lastly, although real-time neonatal seizure prediction has the potential to improve resource allocation in resource-limited countries, we acknowledge that technological availability alone is insufficient to translate into improved clinical practice. As highlighted by Nji et al. regarding AlphaFold's potential to advance biological research in low-resource settings (Nji et al. 2025), concurrent development in research infrastructure and science policies are essential for disseminating technological innovations in these contexts.”*

Also, we have tempered our wording in the Introduction/Background (line 61) by referring to the potential of this technology, as follows: *“In addition, seizure prediction in neonates has the potential to improve neonatal care by optimizing the allocation of EEG monitoring resources, particularly in environments with limited EEG availability…”*

**2. Methods seem sound for training and test sets**

We appreciate this feedback.

**3. Excellent description of SPH and SOP goals.**

We appreciate this feedback.

**4. Wonder if it would be worth doing a subgroup analysis between the two datasets to see if that changes the results - only 2 (4%) with seizures in the cork group.**

We have performed subgroup analysis and have included this in the Results section (line 199):

*“Given the different prevalence of seizures in the subgroups, we performed a subgroup analysis comparing balanced accuracy for the HUH vs Cork datasets (Supplementary Figure 5). This analysis demonstrated that while preictal classification accuracy exceeded chance levels (50%) for most subjects, the performance was relatively higher in the Cork dataset compared to the HUH dataset.”*

**5. How does this short horizon prediction equate to long term seizure risk?**

We have clarified and contrasted this relationship in the Discussion (Line 240), indicating that the short-term predictions should be viewed as complementary information to long-term seizure risk, as follows:

*“our approach offers minute-scale, dynamic predictions that complement these longer-range assessments. By providing more precise, dynamic updates on seizure risk, these short-term predictions may facilitate time-sensitive interventions, such as administration of rapid-acting antiseizure prophylaxis, enable real-time adjustments to resource allocation such as more intensive monitoring.”*

**6. Some concerns about the poor generalizability from the per-subject analyses**

We acknowledge that generalizability may be limited and recommended in the Discussion that future larger-scale external validation studies are warranted (Line 272).

**7. Appropriate discussion of limitations of the study design, results, generalizability**

We appreciate this feedback.

Minor Comments

**8. Would be helpful to have a brief background on who is at risk for these neonatal seizures/encephalopathies - briefly touched on in methods, etc.**

We have added additional context for who is at risk for neonatal seizures to the Methods (Line 91) as follows:

*“We note that seizure etiologies in the HUH dataset included acute events such as encephalitis/meningitis, cerebral infarction, HIE, and intracranial hemorrhage. Together, these comprise acute etiologies which constitute the majority (approximately 75%) of neonatal seizure cases, indicating that our dataset is broadly representative of the neonatal seizure population (Pisani et al. 2021).”*

**Reviewer #2:**

**1. The paper is well written and very detailed, with much of the information found in the supplementary materials. However, I suggest moving some of the tables into the main text for better readability. In particular, the table comparing different methods would be more useful in the main body.**

We appreciate this feedback and have moved Table 1 to the main text, stating: “We evaluated current state-of-the-art DL methods by comparing performance using QEEG features versus automated feature extraction (AFE), (Table 1).”

**2. The 3–7 minute lead time is a major strength because it directly addresses the need for quicker seizure warnings. However, it would help to clearly explain which specific clinical decisions or interventions could be enabled by this short warning window, and how these differ from those triggered by longer-horizon methods.**

In the Discussion (Lines 240-245), we now draw a contrast between short-term vs long-term prediction approaches, and aims as follows: “our approach offers minute-scale, dynamic predictions that complement these longer-range assessments. By providing more precise, dynamic updates on seizure risk, these short-term predictions may facilitate time-sensitive interventions, such as administration of rapid-acting antiseizure prophylaxis, and enable real-time adjustments to resource allocation such as more intensive monitoring.”

In addition, we note that prophylactic ASM, which has been investigated in adult populations could be considered, and state this in the Introduction (line 79) as follows: “potentially improving allocation of monitoring resources and enabling acute therapeutic strategies, such as administration of prophylactic antiseizure therapy[21].”

**3. The authors tested multiple models (ConvLSTM, ResNet, Transformer, etc.) but focused on ConvLSTM. Please add more details on why ConvLSTM outperformed the others. It may help to note that ConvLSTM can capture both short-term features via CNN layers and longer temporal dependencies via LSTM layers.**

We appreciate this insight and elaborate on this further in the Discussion (line 265) as follows: “ConvLSTM incorporates strengths of both convolutional neural network (CNN) and LSTM architectures: the convolutional module extracts short-term temporal features predictive of seizure risk, while the LSTM module learns long-range temporal dependencies.”

**4. The authors mentioned that only the top three QEEG feature categories were used. Please clarify why these three were selected and whether other features would have significantly changed the results.**

These three feature families were selected because of prior study that demonstrated that these three previously had highest performance in feature selection analysis, and have also been independently validated in an external dataset. We clarify this in the Methods (line 121-126) as follows:

*“We previously assessed and ranked a broad set of QEEG features highly predictive of seizure onset using the Boruta feature selection algorithm [26, 27], and here utilize the top three feature categories: standard summary statistics (mean, standard deviation, kurtosis, skew, the 10th percentile, and the 90th percentile)…. The predictive performance of these features have been validated in an external neonatal HIE dataset [17].”*

We also elaborate on the reason to limit the number of features used as follows in the Methods (line 123) as follows: “*We selected only three feature families in order to limit the number of covariates included in the model to reduce the risk of overfitting given our sample size (Harrell et al., 1996).”*

**5. More information is needed on how the authors shaped the data input for ConvLSTM. Please specify the dimensionality of the input and describe any normalization or regularization steps the authors applied.**

We have clarified this in the Supporting Information (Section S2 Methods) as follows:

220 features per 20 second time step were calculated, and the time duration of input was 5 minutes (15 total time steps or 5 * 3 twenty sec epochs), resulting in input tensor of dimensions 220 by 15 per sample. Regularization with drop-out was used, with the specific dropout rate used specified below. Batch normalization was performed where specified.

**6. The dataset appears to be imbalanced. Did the authors consider using weighted losses or focal losses to address this issue? Such methods can help prevent the model from overfitting to the majority class.**

We acknowledge the imbalance in our dataset and clarify that we indeed employed weighted losses, and comment on this in S2 Methods as follows: To address potential bias arising from class imbalance, loss weights were computed separately for each k-fold based on the relative proportions of pre-ictal and interictal segments.

**7. While the authors report the overall false detection rate, it would be helpful to discuss how these false alarms are distributed. For example, do certain subjects experience many more false alarms, or do they occur randomly?**

We have added the distribution of false detections in Supplementary Figure 2b, which indicates that certain subjects did indeed experience relatively high false alarms. Not unexpectedly, this tended to occur in subjects who did not have seizures (these subjects may nonetheless be at risk for seizure, despite not having a seizure during the EEG-recorded period). We have commented on this in the Results (210-213) as follows: “S2b Fig demonstrates that false detections were unevenly distributed, with certain subjects experiencing disproportionately higher false detections. These tended to be subjects without observed seizures, who may nevertheless represent neonates at true risk of seizure despite not having seizures during the recorded period.”

**8. It would be useful to add a brief discussion about how the authors’ approach might be adapted or extended for continuous, real-time monitoring in a clinical setting. This could include how often the model updates its predictions or how it handles continuous data streams.**

We agree that adapting our approach for continuous, real-time monitoring is an important consideration. We elaborate on this in the Discussion as follows (Line 261): “*In addition, our dynamic approach facilitates real-time risk updating, as predictions are updated at 20-second rolling intervals, laying the foundation for adaptation to continuous EEG data streams in clinical settings.”*

**9. Currently, only the code for generating QEEG features is publicly available. Please consider sharing the full data processing and model training code to help other researchers reproduce the results. This would strengthen the paper’s overall impact and transparency.**

We agree and we have shared the model training and main results generation code at <https://github.com/dbernardo05/neoSeer>, and make mention of this in the Methodology.
